# Structural basis of the transcription termination factor Rho engagement with transcribing RNA polymerase from *Thermus thermophilus*

**DOI:** 10.1126/sciadv.ade7093

**Published:** 2023-02-08

**Authors:** Yuko Murayama, Haruhiko Ehara, Mari Aoki, Mie Goto, Takeshi Yokoyama, Shun-ichi Sekine

**Affiliations:** ^1^ Laboratory for Transcription Structural Biology, RIKEN Center for Biosystems Dynamics Research, 1-7-22 Suehiro-cho, Tsurumi-ku, Yokohama 230-0045, Japan.; ^2^ Laboratory for Protein Functional and Structural Biology, RIKEN Center for Biosystems Dynamics Research, 1-7-22 Suehiro-cho, Tsurumi-ku, Yokohama 230-0045, Japan.; ^3^ Graduate School of Life Sciences, Tohoku University, 2-1-1 Katahira, Aoba-ku, Sendai 980-8577, Japan.

## Abstract

Transcription termination is an essential step in transcription by RNA polymerase (RNAP) and crucial for gene regulation. For many bacterial genes, transcription termination is mediated by the adenosine triphosphate–dependent RNA translocase/helicase Rho, which causes RNA/DNA dissociation from the RNAP elongation complex (EC). However, the structural basis of the interplay between Rho and RNAP remains obscure. Here, we report the cryo–electron microscopy structure of the *Thermus thermophilus* RNAP EC engaged with Rho. The Rho hexamer binds RNAP through the carboxyl-terminal domains, which surround the RNA exit site of RNAP, directing the nascent RNA seamlessly from the RNA exit to its central channel. The β-flap tip at the RNA exit is critical for the Rho-dependent RNA release, and its deletion causes an alternative Rho-RNAP binding mode, which is irrelevant to termination. The Rho binding site overlaps with the binding sites of other macromolecules, such as ribosomes, providing a general basis of gene regulation.

## Teaser

Cryo-EM captures the structure of an RNA polymerase elongation complex engaged with the termination factor Rho.

## INTRODUCTION

From bacteria to eukaryotes, DNA-dependent RNA polymerase (RNAP) conducts gene transcription via three sequential processes—initiation, elongation, and termination. The termination process includes the stalling of the transcribing RNAP elongation complex (EC) at termination sites within a gene, followed by RNA and DNA dissociation from RNAP to generate the proper RNA 3′ end. Two termination mechanisms have been elucidated: intrinsic termination that does not require any auxiliary factors and factor-dependent termination that requires one or more specific protein(s) for termination (*1*). In bacteria, the factor-dependent termination is mediated by the transcription termination factor Rho, which terminates transcription at the Rho-dependent terminators present in many genes (*2*–*5*). These two mechanisms can be combined: Rho stimulates termination at some intrinsic terminators (*6*, *7*) and at Rho-dependent terminators after readthrough of the Rho-stimulated intrinsic terminators (*6*). Thus, Rho plays a crucial role in defining the ends of genes and gene boundaries. Besides its basic role in RNA synthesis, emerging evidence has implicated Rho in key regulatory mechanisms, including transcription suppression of untranslated mRNA (the polarity effects) (*8*) and small RNA or riboswitch-dependent regulation (*9*, *10*). Rho-dependent termination is also implicated in “housekeeping” mechanisms, such as suppressing pervasive antisense transcription (*11*), silencing horizontally transferred genes (*12*), and resolving conflicts between transcription and replication (*13*).

Rho is an adenosine triphosphate (ATP)–dependent RNA translocase/helicase with 5′ → 3′ directionality (*14*, *15*). It consists of the N-terminal domain (NTD) and the C-terminal domain (CTD), which have the primary RNA binding site (PBS) and the secondary RNA binding site (SBS), respectively (*16*–*18*). Rho forms a ring-shaped homo-hexameric complex, mediated by the CTD (*18*–*20*). The Rho hexamer can adopt both open- and closed-ring conformations. When Rho binds RNA and ATP, it assumes the closed-ring conformation to thread the RNA into its central channel composed of the SBS (*21*). At the Rho-dependent terminator, Rho engages the nascent RNA emerging from the EC and dissociates the RNA and DNA from RNAP (*22*–*24*). However, the mechanism by which Rho interacts with RNAP to cause termination remains elusive.

Cryo–electron microscopy (cryo-EM) structures of *Escherichia coli* RNAP bound with Rho, and transcription elongation factor NusA and/or NusG were recently reported (*25*, *26*). In these structures, the Rho hexamer was in an open conformation without engagement with RNA and bound the RNAP via an *E. coli–*specific insertion domain (βSI2) and NusA. These structures suggested that Rho induces an allosteric change in RNAP to destabilize the EC for priming the dissociation of RNA and DNA. However, these studies also postulated that termination ultimately involves the Rho ring closure and its engagement with RNA, followed by the Rho translocation to release RNA and DNA. In this study, we recapitulated the Rho-associated EC using proteins from the bacterium *Thermus thermophilus* and performed structure determination by cryo-EM single-particle analysis. The structure revealed the manner by which the nascent RNA-engaged Rho hexamer in the closed-ring conformation interacts with the RNA exit site of RNAP, illuminating the EC state before termination.

## RESULTS

### Rho-dependent RNA release assay

**Fig. 1.**
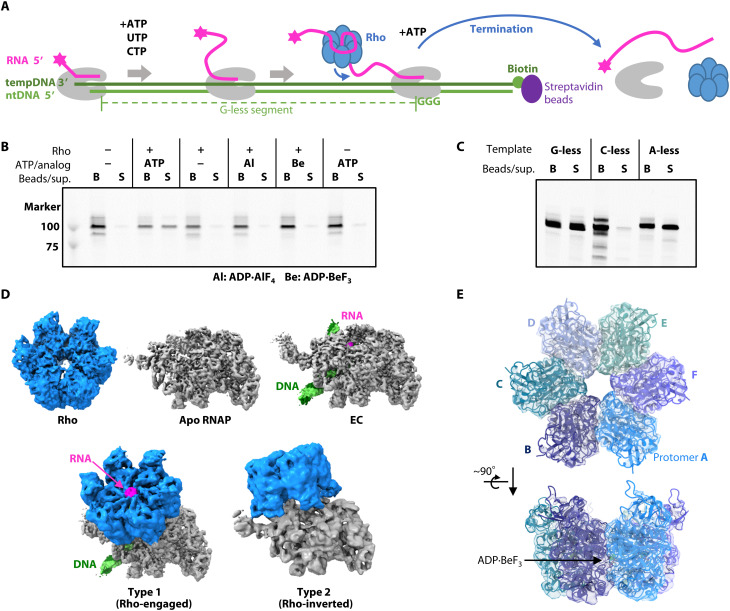
Reconstitution and cryo-EM analysis of the Rho-EC complex. (**A**) Schemes of the Rho-EC reconstitution and the RNA release assay. (**B**) Rho-dependent RNA release assay. Rho and ATP (or ADP·AlF_4_ or ADP·BeF_3_) were added to ECs immobilized on streptavidin magnetic beads, and the mixture was incubated for 10 min at 65°C. Each sample was separated into bead (B) and supernatant (S) fractions and analyzed by urea–polyacrylamide gel electrophoresis (PAGE). RNA was detected by its 5′-fluorescent label. (**C**) RNA release assay with template DNA variants. ECs were prepared using G-less, C-less, or A-less template DNA immobilized on streptavidin magnetic beads. Rho and ATP were added to the ECs, and the mixture was incubated for 5 min at 65°C. (**D**) Structures of the complexes contained in the cryo-EM sample. Cryo-EM maps of Rho, apo RNAP, EC, and type 1 and type 2 complexes are shown. The maps are colored to match the structural models (gray, RNAP; blue, Rho; green, DNA; magenta, RNA). (**E**) Structure of *T. thermophilus* Rho. The Rho cartoon model is shown in two orientations, overlaid with a transparent cryo-EM map.

To examine the Rho-dependent RNA release activity from the RNAP EC, we developed an in vitro experimental system using *T. thermophilus* Rho and RNAP. A 199–base pair (bp) DNA containing a 114-bp G-less segment was designed, and one end of the DNA was immobilized on a magnetic bead (Fig. 1A and fig. S1A). The other end of the DNA has a 3′ overhang of the template strand, to which a fluorescently labeled primer RNA was annealed, and then RNAP was loaded to assemble the EC (*27*). A 125-nucleotide RNA was elongated by adding ATP, cytidine triphosphate (CTP), and uridine triphosphate (UTP), and the EC was stalled at the first G stretch on the DNA. After the nucleotide substrates were removed, Rho was loaded to form the EC-Rho complex. When ATP was added, the RNA was released from the immobilized complex to the solvent, indicating the termination proficiency of this complex (Fig. 1B). The RNA release is ATP dependent, as it did not occur in either the absence of ATP or the presence of an ATP analog, adenosine diphosphate (ADP)–aluminum fluoride (ADP·AlF_4_) or ADP–beryllium fluoride (ADP·BeF_3_).

To investigate the sequence dependence of the Rho-dependent RNA release, we performed RNA release assays with various template DNAs, in which the G-less segment was modified. The C-less template was constructed by replacing C in the G-less segment with G (fig. S1A). The A-less template was also constructed by replacing A in the G-less segment by G. The RNA release with the C-less template was deficient (Fig. 1C), suggesting that, similar to *E. coli* Rho, *T. thermophilus* Rho also prefers C-rich sequences (*28*).

We also examined the effects of transcription elongation factors in the Rho-dependent RNA release. In *E. coli*, the transcription elongation factor NusG directly binds Rho and enhances Rho-dependent termination (*29*). Despite its high sequence and structural similarity to *E. coli* NusG, *T. thermophilus* NusG reportedly does not bind Rho (*30*). *T. thermophilus* NusG did not strongly bind the EC in our in vitro assay, as the majority of NusG was dissociated from the EC after washing the beads (fig. S1B). NusG had no effect on the Rho-dependent RNA release (fig. S1C), and therefore, we omitted NusG in this study. In our in vitro transcription system, GreA, which helps to reactivate a backtracked EC (*31*), was included to suppress the EC stalling before reaching the termination site. The interaction between GreA and the EC is relatively weak, as the majority of GreA was dissociated from the EC after washing the beads (fig. S1B). In addition, GreA did not affect Rho-dependent RNA release (fig. S1D).

### Cryo-EM analysis of the RNAP-Rho complexes

For the cryo-EM analysis of the EC-Rho complex, the EC was prepared using the same DNA/RNA as in the RNA release assay. The nucleotide substrates and RNAP-unbound DNA/RNA were removed from the EC by gel filtration chromatography (fig. S2A). The purified EC was mixed with Rho in the presence of ADP·BeF_3_ and cross-linked with BS3 to stabilize the complex before the preparation of cryo-grids. Approximately 33,000 cryo-EM images were collected for single-particle analysis (fig. S2B and table S1). By the initial two-dimensional (2D) classification, the particles were classified into the Rho-only and RNAP-containing classes (Fig. 1D and fig. S2, C and D). The Rho structure was determined at 3.5-Å resolution (Fig. 1D and fig. S3). The Rho hexamer assumes a closed-ring conformation, with the ATP analog ADP·BeF_3_ bound at the interface between the protomers (Fig. 1E and fig. S2E). The closed-ring structure is similar to the previously reported crystal structure of the *E. coli* Rho closed hexamer bound with ADP·BeF_3_ and RNA (C_α_ root mean square deviation = 1.9 Å; fig. S2F) [Protein Data Bank (PDB) 5JJI] (*20*).

**Fig. 2.**
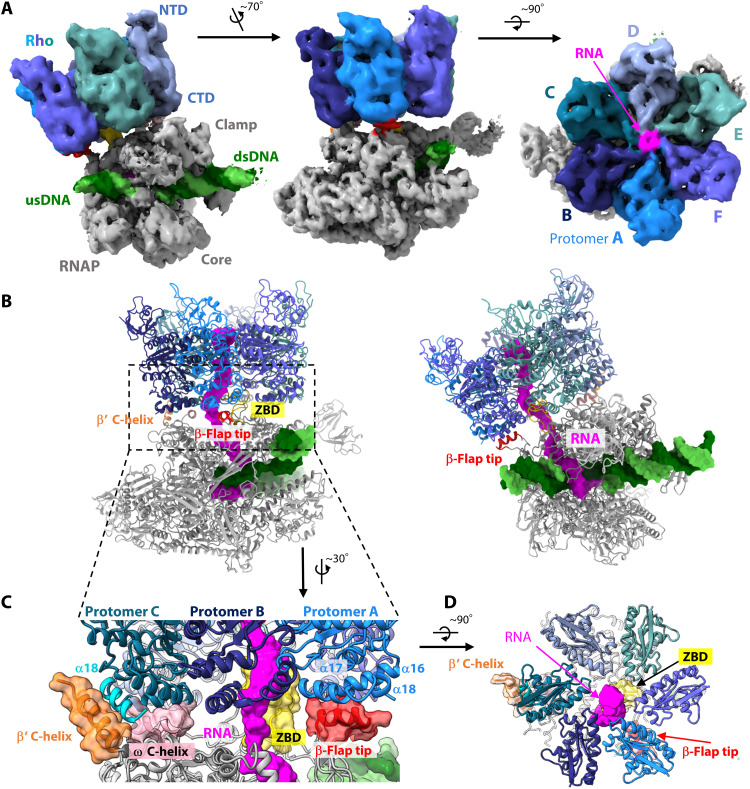
Structure of the Rho-engaged EC (type 1). (**A**) Cryo-EM map of the Rho-engaged EC (the type 1 complex) in three orientations. The composite map was generated from the EC-masked (3.7-Å resolution) and Rho-masked (4.6-Å resolution) focused maps by Phenix (*53*). The map is colored to match the model colors: Rho is colored as in Fig. 1D, RNAP is colored gray [β-flap tip, β′ C-helix, and zinc binding domain (ZBD) in red, orange, and yellow, respectively], the template DNA is colored dark green, nontemplate DNA is colored light green, and RNA is colored magenta. usDNA, upstream DNA; dsDNA, downstream DNA. (**B**) Structure of the type 1 complex. The structural model is displayed in two orientations, with the protein shown in a cartoon representation and the DNA/RNA shown in a surface representation. (**C**) Close-up views of the RNAP-Rho interface. Transparent surfaces of β-flap tip, ZBD, β′ C-helix, and ω C-helix domains are overlaid. (**D**) The Rho hexamer ring from the NTD side.

The particles containing RNAP were further sorted by 3D classification into DNA/RNA-unbound RNAP (apo RNAP), EC, and two forms of RNAP-Rho complexes (referred to as the type 1 and type 2 complexes) (figs. S3 and S4). The EC holds a transcription bubble containing a 10-bp DNA/RNA hybrid within the RNAP main channel (Figs. 1D and 2). The EC is stalled immediately before the G-triad of the template DNA (Fig. 1A). The type 1 and type 2 RNAP-Rho complexes both revealed the closed-ring Rho hexamer with RNA threaded in the central channel, but they have different Rho binding sites and orientations relative to RNAP. In the type 1 complex, Rho is bound to the RNA exit site of the EC via its CTD, thus representing the Rho-engaged state of the EC. By contrast, in the type 2 complex, Rho is bound to apo RNAP via its NTD, and this probably represents a nonproductive state. More particles of the type 2 complex than the type 1 complex were obtained.

### Structure of the Rho-engaged EC (type 1)

In the type 1 complex, the Rho hexamer assumes a closed-ring conformation and is engaged with the EC (Fig. 2 and movie S1). Rho directly binds the RNAP RNA exit site via its CTD side, and thus, the Rho ring surrounds the exiting nascent RNA to form a continuous RNA path from the RNAP RNA exit channel to the Rho central channel. The CTD side of the Rho hexamer is widened as compared with that of the free Rho hexamer (fig. S5A). The nascent RNA emerging from RNAP is seamlessly threaded into the Rho central channel (SBS). On the NTD side of the Rho hexamer, the RNA extends straight out of the central channel (fig. S5B). Although our RNA release assay revealed the preference of the C-rich sequence by *T. thermophilus* Rho (Fig. 1C), no clear RNA density was observed at the PBS of each Rho protomer. A sequence comparison indicated that three (Arg^66^, Asp^78^, and Tyr^80^) of the six residues that recognize the pyrimidine nucleotides in *E. coli* Rho (PDB 1PVO) (*16*, *18*) are different in *T. thermophilus* Rho (fig. S5C). Therefore, one possible explanation for the absence of RNA density in the PBS is that the RNA binding by *T. thermophilus* Rho might be weaker than that by *E. coli* Rho. Alternatively, the C-rich sequence may only be required for the Rho loading step and dispensable after the Rho ring closure and engagement with the EC.

The β-flap domain of the RNAP β subunit (residues β762 to β784) forms part of the RNA exit channel. The tip helix of the β-flap domain is reoriented and adapted to the C-terminal side of a Rho protomer (protomer A in Fig. 2), contacting helices α16 (residues 381 to 395), α17 (residues 399 to 412), and α18 (residues 415 to 419). Thereby, the position of the β-flap tip helix is slightly farther away from the exiting RNA, as compared with its position in the previously reported crystal structure (PDB 2O5I) (fig. S5D) (*32*). The zinc binding domain of the β′ subunit (residues β′56 to β′80), which is also part of the RNA exit channel, enters the central channel of the Rho ring, similar to an axle of a wheel. Rho also binds to the C terminus of the β′ subunit and the ω subunit of RNAP. The C-terminal helix (helix α18; residues 415 to 426) of one Rho protomer (protomer C) is longer than those in the other protomers (residues 415 to 420) and forms a helix bundle with the C-terminal helix of the RNAP β′ subunit (residues β′1489 to β′1502). The formation of this helix bundle displaces the C-terminal helix of the RNAP ω subunit (residues ω82 to ω92), which instead contacts the bottom part of the Rho protomer C (Fig. 2D and fig. S5E). Thus, the nascent RNA, emerging between the zinc binding and β-flap domains, is seamlessly directed to the central channel and passes through the narrowest part consisting of the SBSs of the Rho protomers (Fig. 2). The observed Rho-EC binding mode is similar to that in the recently reported *E. coli* Rho-EC complex structure (fig. S6) (*33*).

### Critical role of the β-flap tip in the Rho-dependent RNA release and the type 1 complex formation

**Fig. 3.**
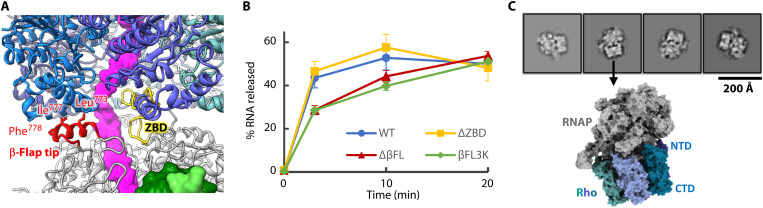
Rho-dependent RNA release assays with RNAP variants. (**A**) Close-up view of the Rho-RNAP interface around the β-flap and ZBD. The three hydrophobic residues substituted for lysine in the βFL3K variant are shown in stick models. (**B**) Rho-dependent RNA release activity examined with RNAP variants. Mean values of three independent experiments were plotted (error bars = SD) (table S2). WT, wild type. (**C**) 2D class averages of ~3000 particles of the RNAP-Rho complex prepared using the Δβ-flap tip RNAP variant. A surface model of the type 2 complex aligned with a 2D class average is shown below.

To investigate the importance of the Rho-contacting RNAP domains in transcription termination, we created mutant RNAPs lacking the β-flap tip or the zinc binding domain and examined their Rho-dependent RNA release activities. While the deletion of the zinc binding domain did not affect the RNA release activity, the absence of the β-flap tip impaired the activity (Fig. 3, A and B, and fig. S7). In addition to the deletion of the β-flap tip, we examined mutations of hydrophobic residues in the β-flap tip, which could directly contact the C-terminal side of Rho (L773K/I777K/F778K and βFL3K). This triple mutant impaired the RNA release activity similarly to the β-flap tip deletion mutant. Thus, the β-flap tip is crucial for the Rho-dependent RNA release.

We also performed a cryo-EM analysis of the EC-Rho complex prepared using the β-flap tip-deleted RNAP. The structure analysis yielded only the type 2 complex class (Fig. 3C). This suggests that the β-flap tip plays critical roles in the type 1 complex formation through interactions with the C-terminal part of Rho and in the functions of the type 1 complex during termination.

### Structure of RNAP bound with an inverted Rho (type 2)

**Fig. 4.**
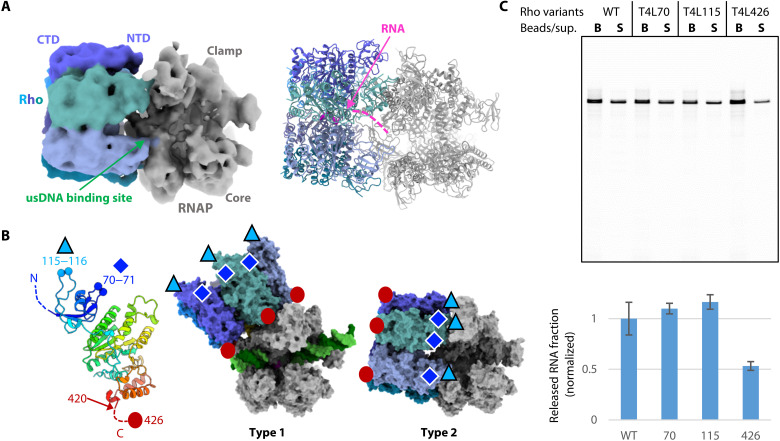
Structure of RNAP bound with an inverted Rho (type 2). (**A**) Structure of the type 2 complex. The 5.8-Å cryo-EM map (left) and the structural model (right). The map is colored as in Fig. 2. (**B**) T4 lysozyme (T4L) insertion sites. Left: The T4L insertion positions in the Rho_T4L variants are mapped on the Rho protomer structure. Middle and right: The surface models of the type 1 and type 2 complexes are shown with the same RNAP orientation. The T4L positions are marked on the structures. (**C**) The RNA release assay with the T4L-inserted Rho variants. Wild-type or T4L-inserted Rho was added to immobilized ECs and incubated at 35°C for 10 min. Top: A gel image for the RNA release assay. Bottom: Mean values of three independent experiments are shown, normalized to the mean value with the wild-type Rho as 1 (error bars = SD) (table S2).

In the type 2 complex, Rho is bound to RNAP in an inverted orientation, as compared with that in the type 1 complex (Fig. 4A). The Rho hexamer assumes a closed-ring conformation with RNA threaded through the Rho central channel and binds near the DNA exit site (*34*) of RNAP through its NTD side. The RNAP adopts an open-clamp conformation, which resembles that in the apo RNAP rather than the EC (fig. S4D). While the RNAP main channel is empty, with no bound DNA/RNA hybrid, the Rho-bound RNA appears to enter and interact with the upstream DNA binding site of the RNAP (fig. S8). Four of the six Rho protomers are involved in the RNAP interaction: Rho protomers B and C contact the base part of the β-flap domain (residues β727 to β738), protomers C and D contact the jaw-lobe 1 domain, and protomer E contacts the clamp coiled-coil of RNAP.

To investigate whether the inverted binding mode of Rho in the type 2 complex is important, we created mutant Rho proteins in which T4 lysozyme (T4L) is inserted into the Rho NTD (Rho_T4L70 and Rho_T4L115) (Fig. 4B). As the bulky T4L insertion would intervene between the Rho NTD and RNAP, it is expected to hinder the inverse RNAP-Rho interaction observed in the type 2 complex but not the RNAP-Rho interaction of the type 1 complex. These T4L-inserted Rho mutants showed RNA release activities comparable to that of wild-type Rho (Fig. 4C). We also created a Rho mutant in which T4L is appended to the Rho C terminus (Rho_T4L426). For this mutant, T4L would interfere with the RNAP-Rho interaction in the type 1 complex but not that in the type 2 complex. The Rho_T4L426 mutant exhibited impaired RNA release activity (Fig. 4C). These results again support the proposal that the type 1 complex is essential for the RNA release, but the type 2 complex is not.

## DISCUSSION

In this study, we determined the cryo-EM structure of a Rho-engaged EC from *T. thermophilus* (the type 1 complex). In this structure, the Rho hexamer is in a closed-ring conformation and engaged with the RNA and EC. The Rho hexamer binds to the rim of the RNA exit channel of EC via its CTD side and seamlessly threads the emerging nascent RNA into its central channel. Our results demonstrated that the β-flap tip, which forms part of the RNA exit channel, is essential for the Rho-EC engagement and the Rho-dependent RNA release. Thus, Rho binding by the EC is mediated by both the nascent RNA and the specific Rho-RNAP interaction, consistent with the “classic” view of Rho-dependent transcription termination (*1*–*5*).

Our cryo-EM analysis also provided another Rho-RNAP complex (the type 2 complex), in which Rho binds near the DNA exit site of apo RNAP through its NTD side. The T4L insertions into the NTD did not affect the RNA release activity of Rho. Moreover, the deletion of the RNAP β-flap tip, which is critical for the Rho-dependent RNA release, enhanced the formation of the type 2 complex. Therefore, the inverted Rho-binding mode in this complex is unlikely to be relevant to termination. The abundance of the type 2 complex in the cryo-EM particles suggests that Rho may preferentially bind apo RNAP in this alternative mode. Therefore, this complex could potentially resemble a posttermination state formed after the EC has released DNA and RNA.

In previous studies of *E. coli* Rho-RNAP complex structures, Rho was bound near the DNA exit site of the EC through its NTD side, as in our inverted Rho-RNAP structure (the type 2 complex) (fig. S9) (*25*, *26*). However, in the *E. coli* complexes, the Rho hexamer assumes an open-ring conformation without full RNA engagement, and the Rho-RNAP interaction is mediated by an *E. coli*–specific insertion (βSI2) of RNAP and NusA. While the authors of these studies postulated that the Rho-RNAP interaction induces allosteric changes in the EC active site, they also proposed that transcription termination occurs via its subsequent state, in which Rho assumes a closed-ring conformation to gain access to both the nascent RNA and the RNA exit channel of RNAP. Our current structure of the Rho-engaged EC (the type 1 complex) might have captured this state.

The Rho-RNAP binding mode in the current Rho-engaged EC structure is also consistent with the NusG-dependent Rho recruitment. The transcription elongation factor NusG recruits Rho to the EC and facilitates transcription termination in *E. coli* (*4*) and *Bacillus subtilis* (*6*). The Rho CTD becomes the interface with the NusG CTD, and their interaction promotes termination (*35*). Although *T. thermophilus* NusG did not bind either the EC or Rho, modeling of an *E. coli* EC-Rho complex based on the current type 1 complex structure suggests that the NTD and CTD of NusG could bridge between RNAP and Rho, respectively, reasonably explaining the NusG-mediated recruitment of Rho to the EC (fig. S10) (*35*, *36*). This model is consistent with the recently reported structure of the *E. coli* EC-Rho-NusG complex (*33*).

How does Rho terminate transcription? If Rho “pulls” RNA by its ATP-dependent RNA translocation activity, as suggested previously, then it would perturb the DNA/RNA hybrid within the transcription bubble or the RNAP-DNA/RNA contacts (*22*, *23*). This would trigger the destabilization of the EC, leading to the RNAP conformational change including the clamp opening, the bubble collapse, and the release of DNA/RNA from the RNAP (*37*). When the apo RNAP structure is superimposed with the Rho-engaged EC structure, the opened clamp in apo RNAP fits with the bottom parts of the Rho hexamer without severe steric clash (protomers E and F). Therefore, the Rho-engaged EC is compatible with the clamp opening of RNAP for the release of the bound DNA/RNA (fig. S11).

This study revealed that Rho binds directly to the RNA exit site of RNAP. In some bacteria including *E. coli*, RNAP interacts with the trailing ribosome to establish the transcription-translation coupling (*38*–*41*). The transcription elongation factors NusG and NusA mediate this interaction, facilitating dynamic coupling (*40*, *41*). Once the ribosome reaches the stop codon and detaches from the preceding RNAP, Rho can access the RNAP to terminate transcription. The ribosome binding site of RNAP overlaps with the Rho binding site, suggesting that their RNAP bindings are mutually exclusive (fig. S12). This reasonably explains the coordination of transcription and translation in gene regulation. The Rho binding site also overlaps with that for the antitermination complex (*42*, *43*), explaining how the latter blocks the Rho access to the RNAP transcribing ribosomal genes or phage operons. In contrast, transcription and translation are uncoupled in *B. subtilis*, where Rho-dependent termination is highly dependent on cis elements, such as the *rut* sites (*44*, *45*). Recent studies have revealed that *B. subtilis* Rho stimulates termination at intrinsic terminators with weaker motifs (*6*, *46*). Our current structure (type 1) suggests that Rho could participate in this process by resolving the RNA structures formed upstream of the termination site. Thus, the current structure provides the foundations of gene regulation via the coordination or competition of multiple molecular processes.

## MATERIALS AND METHODS

### Proteins

For the expression of recombinant *T. thermophilus* RNAP, the genes encoding the RNAP subunits were cloned into the pETDuet-1 (β and β′ subunits in MCS1, α subunit in MCS2, and a FLAG tag added to the C-terminal end of the β′ subunit) and pET-47b (ω subunit) vectors. These plasmids were coexpressed in *E. coli* Rosetta2 (DE3). Plasmids for the expression of RNAP variants were constructed on the basis of these plasmids. In ΔβFL and ΔZBD, the β-flap tip helix (residues β762 to β782) and the zinc binding domain (residues β′57 to β′80), respectively, were removed, and the gaps were filled with three alanine residues. In βFL3K, residues Leu^773^, Ile^777^, and Phe^778^ of the β subunit were replaced by lysine residues. The recombinant RNAP variants were purified as described previously (*31*).

The gene encoding *T. thermophilus* Rho was cloned into the pET-47b vector. The N-terminally His-tagged Rho was expressed in *E. coli* Rosetta2 (DE3). The cells were suspended in lysis buffer A [50 mM Hepes-NaOH (pH 7.5), 500 mM KCl, 10% glycerol, and 1 mM dithiothreitol (DTT)] and disrupted by sonication. The lysate was heated at 70°C for 10 min to denature the *E. coli* proteins, and the precipitate was removed by centrifugation. The supernatant was loaded onto a Ni affinity column (Ni-Sepharose 6 FF, Invitrogen). The column was washed with lysis buffer A supplemented with 10 mM imidazole. The His-tag was cleaved by human rhinovirus (HRV) 3C protease on the column for 16 hours at 4°C. The flow through fraction after the HRV 3C cleavage was purified on a gel filtration column (Superdex 200, Cytiva) equilibrated with buffer A.

The Rho variants with a T4L insertion (Rho_T4L70 and Rho_T4L115) were constructed by inserting the T4L gene with a two-residue spacer (Gly-Ser) on both ends between residues Gln^70^ and Asp^71^ (Rho_T4L70) or Glu^115^ and Asn^116^ (Rho_T4L115) of the Rho gene. The Rho mutant with T4L attached to the C terminus (Rho_T4L426) was constructed by connecting the T4L gene downstream from the C terminus (Arg^426^) of the Rho gene. The Rho_T4L variants were cloned into the pET-47b vector, and the proteins were expressed and purified similarly to the wild-type Rho, except that the lysate was not heat-treated.

The gene encoding *T. thermophilus* GreA was cloned into the pET-22b vector. The C-terminally His-tagged GreA was expressed in *E. coli* Rosetta2 (DE3). The cells were disrupted in lysis buffer B [50 mM tris-HCl (pH 8.0), 150 mM NaCl, and 1 mM DTT). The lysate was heated at 80°C for 10 min, and the supernatant was loaded onto a Ni affinity column (Ni-Sepharose 6 FF, Invitrogen). GreA was eluted with lysis buffer supplemented with 500 mM imidazole and then dialyzed against lysis buffer B.

The gene encoding *T. thermophilus* NusG was cloned into the pET-47b vector. The N-terminally His-tagged NusG was expressed in *E. coli* Rosetta2 (DE3). The cells were disrupted in lysis buffer B. The lysate was heated at 80°C for 10 min, and the supernatant was loaded onto a Ni affinity column (Ni-Sepharose 6 FF, Invitrogen). The His-tag was cleaved by HRV 3C protease on the column, and NusG was eluted with lysis buffer B.

### Rho-dependent termination assay

The EC of RNAP was prepared by walking the RNAP on polymerase chain reaction (PCR)–amplified DNA templates (Fig. 1A and fig. S1A). The template DNA sequence was synthesized using primers T1_Fw (covers positions −22 to +48, with the transcription start position as +1), T2_Rv (+32 to +103), T3_Fw (+89 to +156), and T4_Rv (+140 to +171) (fig. S1A), and the resulting ~200-mer DNA was cloned between the Bam HI and Xho I sites of the pETDuet-1 vector. The biotinylated template DNA was PCR-amplified using primers T0_fw (−22 to +9) and Bio_Rv (anneals to positions +136 to +156 of the template DNA, GAACGCATTACCAGAGAATTCACGGGAAAGTCGACAGGGATC, 5′-biotinylated).

About 1 pmol of the biotinylated template DNA was captured on the streptavidin magnetic beads (Dynabeads MyOne Streptavidin C1, Invitrogen) in buffer A [40 mM tris-HCl (pH 7.4) and 1 M NaCl]. The DNA was digested by Pst I for 1 hour at 37°C in H buffer [50 mM tris-HCl (pH 7.5), 100 mM NaCl, 10 mM MgCl_2_, and 1 mM DTT] to make a 3′ protrusion at the upstream DNA end. After digestion, the beads were washed twice with M buffer [50 mM tris-HCl (pH 7.5), 50 mM NaCl, 10 mM MgCl_2_, and 1 mM DTT]. For the EC reconstitution, 100 nM RNAP, 150 nM fluorescently labeled primer RNA (AAUUUGCAGGA; 5′-DY647 labeled), 200 nM GreA, and 100 μM NTP substrates (ATP/CTP/UTP for the G-less template, ATP/GTP/UTP for the C-less template, and CTP/GTP/UTP for the A-less template) were added to the beads, and the mixture was incubated for 15 min at 65°C in 10 μl of M buffer. The beads were washed three times with M buffer, and the remaining EC was suspended in 4 μl of M buffer and preincubated for 1 min at 35° or 65°C for the subsequent Rho-dependent termination assay. For termination, 1 μl of a mixture of Rho (final 200 nM) and ATP (final 1 mM) was added to the EC on the beads. At the indicated time point, the supernatant was separated from the beads by standing the tubes for 30 s on a magnetic stand. For electrophoresis, 5 μl of urea–polyacrylamide gel electrophoresis (PAGE) sample buffer [50 mM tris-HCl (pH 7.5), 50 mM EDTA, 5 M urea, and 1% orange G] was mixed with the sample, and the RNAs were fractionated by 10 or 15% PAGE with gels containing 7 M urea. The fluorescent-labeled RNA was imaged and quantitated with an ImageQuant LAS-4000 (GE Healthcare) or Typhoon (Amersham) imager. The same reaction was performed in the absence of ATP or in the presence of ATP analogs. The ATP mimic ADP·AlF_4_ was prepared by mixing ADP, AlCl_3_, and NaF in a molar ratio of 1:5:20. The ATP mimic ADP·BeF_3_ was prepared by mixing ADP, BeSO_4_, and NaF in a molar ratio of 1:5:15.

### EC-Rho preparation for cryo-EM

The RNAP, Pst I–digested template DNA, and primer RNA were mixed at a molar ratio of 1:1.5:2 in H buffer. RNAP was walked on the template DNA by adding the ATP/UTP/CTP substrate and GreA. The mixture was incubated for 20 min at 65°C and then loaded onto a Superose 6 gel filtration column (Cytiva). Fractions containing RNAP, DNA, and full-length RNA were collected (fig. S2A); concentrated; and exchanged to buffer C [20 mM Hepes-NaOH (pH 7.5), 250 mM KCl, 5 mM MgCl_2_, and 1% glycerol), and protein amounts were estimated by SDS-PAGE. Approximately 20 pmol of the EC was mixed with 25 pmol of Rho in the presence of 0.5 mM ADP·BeF_3_, in 400 μl of buffer C. The sample was cross-linked with 3 mM BS3 (Thermo Fisher Scientific) for 30 min at 30°C. The cross-linking was quenched by adding 100 mM tris-HCl (pH 7.5). For the grid preparation, the samples were concentrated to about 20 μl. The samples were applied to Quantifoil R1.2/1.3 Copper 300 mesh grids (Quantifoil Micro Tools) and were plunge-frozen with an EM GP2 (Leica) at 10°C and 80% humidity.

### Cryo-EM data collection and image analysis

Cryo-EM images were recorded using a Krios G4 transmission electron microscope (Thermo Fisher Scientific) equipped with a BioQuantum energy filter and a K3 direct electron detector (Gatan) (fig. S2B). Two datasets of ~13,000 and ~20,000 micrographs were collected (table S1). Movies were aligned and dose-weighted using Relion3 (*47*). Contrast transfer function (CTF) estimation was performed by gctf (*48*). Particle picking was performed with Warp (*49*) and Topaz (*50*), and the particle coordinates from each program were merged after 2D classification.

Approximately 1,641,000 particles of RNAP and RNAP-Rho complexes were selected by 2D classification (fig. S2C) and subjected to 3D classification to discard particles without RNAP. The remaining ~997,000 particles were subjected to 3D refinement using an RNAP mask, followed by Bayesian polishing, local CTF estimation, and beam tilt refinement to improve the resolution. The particles from two datasets were then merged for further 3D classification, which revealed the classes of apo RNAP, EC, and RNAP-Rho complexes 1 and 2 (fig. S3). For this classification, a multitemplate reference including type 1 and type 2 complexes, EC, and apo RNAP templates, created using maps obtained from preliminary classifications, was used for better discrimination between the complexes.

For the type 1 complex, the initial ~112,000 particles were refined with an overall mask and then subtracted with a mask around Rho. Several rounds of 2D and 3D classifications were performed to exclude particles with poor Rho density (fig. S4A). Approximately 43,000 particles with clear Rho density were selected for the final reconstruction of a 4.0-Å map. As the quality of the overall reconstruction was compromised by the flexibility between Rho and the EC, independent reconstructions using tight masks around EC or Rho were also performed, respectively, and used for model building (fig. S4A).

For the type 2 complex, ~269,000 particles from the initial 3D classification were further classified using a mask around the type 2 complex (fig. S4B). A 5.8-Å map was reconstructed using ~147,000 particles.

About 612,000 particles of EC and apo RNAP were reclassified through a 3D classification using a reference including the EC and apo RNAP templates. The selected ~220,000 EC particles and ~392,000 apo RNAP particles yielded 3.2- and 3.4-Å maps, respectively (fig. S4C).Approximately 693,000 Rho-hexamer particles were selected by 2D classification (fig. S2D) and subjected to 3D classification (fig. S3). Subsequently, ~239,000 particles were selected for 3D refinement with C6 symmetry enforced and subsequent Bayesian polishing. The particles were further 3D classified in C1 symmetry with symmetry relaxation by C6. About 205,000 particles were subjected to 3D refinement with C6 symmetry. Local CTF estimation and beam tilt refinement were performed to obtain a final 3.5-Å map.

### Model building

The structure model of the *T. thermophilus* Rho protomer was generated by homology modeling, using the *E. coli* Rho crystal structure (PDB 5JJI) (*20*) as the template and the SWISS-MODEL server (*51*, *52*). The model of Rho protomers excluding 59 residues at the N terminus was fitted into the cryo-EM map of EC-unbound Rho and refined with Phenix real space refinement (*53*). For the model building of the Rho-unbound EC and apo RNAP, the crystal structure of the *T. thermophilus* EC (PDB 2O5I) (*32*) was fitted into the cryo-EM maps, and the models were refined with Phenix real space refinement. Models of RNAP-Rho complexes were generated by fitting the coordinates of the EC-unbound Rho and the Rho-unbound EC/RNAP into the maps. In the type 1 complex, structure models of the RNAP-Rho interface (the β-flap tip, the β′ C-helix, the ω C-helix of RNAP, and the α18 helix of Rho protomer C) were built into the cryo-EM map using Coot (*54*) and then refined with Phenix. All structure figures were created using ChimeraX (*55*).

## Notes

View/request a protocol for this paper from *Bio-protocol*.
