# Supplementary material for: Structural basis of the transcription termination factor Rho engagement with transcribing RNA polymerase from Thermus thermophilus
Source: Sci Adv. 2023 Feb 8;9(6):eade7093. doi: 10.1126/sciadv.ade7093 (PMC9908020; doi:10.1126/sciadv.ade7093)
Supplement: Supplementary file 1 — Figs. S1 to S12 Tables S1 and S2 [file sciadv.ade7093_sm.pdf]

Science Advances
AAAS

Supplementary Materials for

Structural basis of the transcription termination factor Rho engagement with transcribing RNA polymerase from Thermus thermophilus

Yuko Murayama *et al.*

Corresponding author: Shun-ichi Sekine, shunichi.sekine@riken.jp

Sci. Adv. **9**, eade7093 (2023)
DOI: 10.1126/sciadv.ade7093

The PDF file includes:

Figs. S1 to S12
Tables S1 and S2
Legend for Movie S1

Other Supplementary Material for this manuscript includes the following:

Movie S1

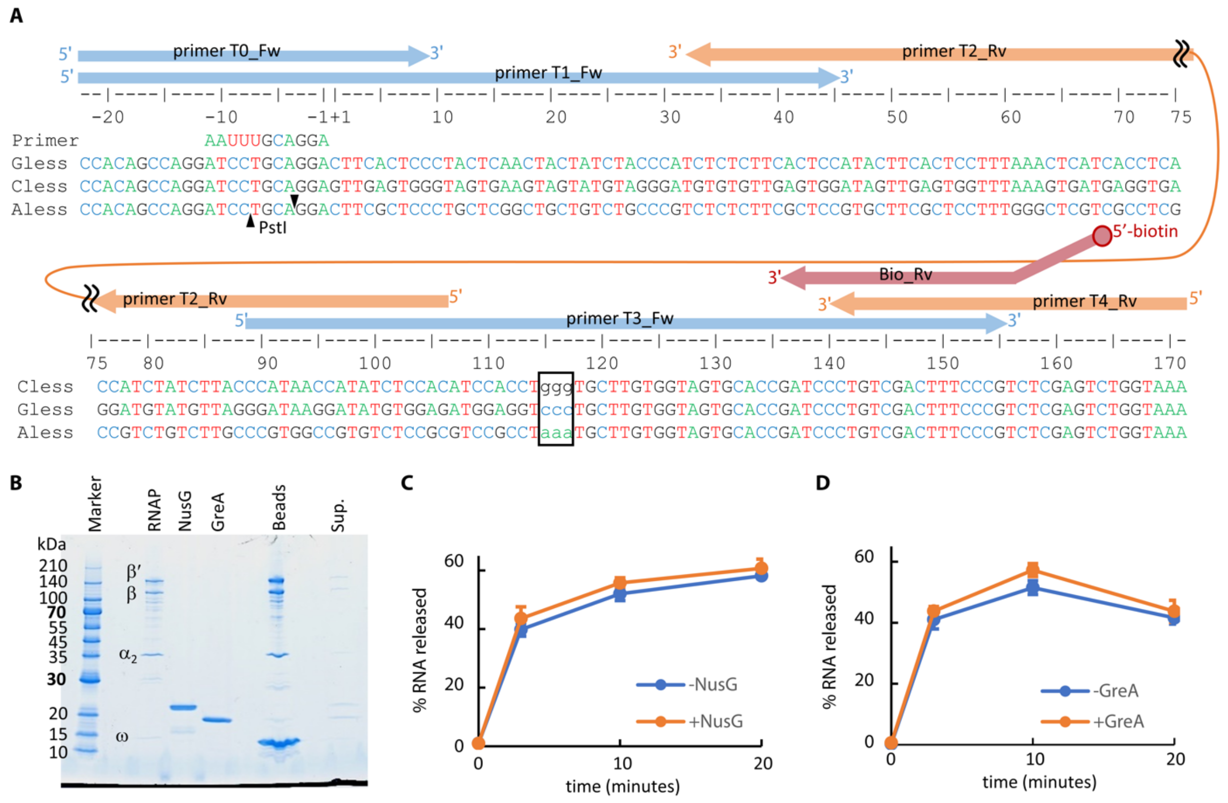

Fig. S1. Rho-dependent RNA release assay.

(A) A schematic representation of the DNA and RNA for EC preparation. Sequences of the sense (non-template) strand of DNA variants and the primer locations are shown.

(B) NusG and GreA do not bind tightly to the EC. The PstI-digested template DNA (2 pmol) was mixed with 10 µl of magnetic bead solution in buffer A. After an incubation for 15 minutes at room temperature, the beads were washed three times with M buffer. Afterwards, 2 pmol of RNAP, 4 pmol of GreA, 4 pmol of NusG, and 2 nmol of substrates ATP/UTP/CTP were added to the beads in 40 µl of M buffer, and the mixture was incubated for 15 minutes at 65˚C. Aliquots (10 µl) of the supernatant fraction and the entire bead fraction after three washes with M buffer were analyzed by SDS-PAGE.

(C) RNA release assay with NusG. The bead-immobilized EC was prepared without NusG. NusG (2 pmol per sample) or buffer was added to the EC and incubated at room temperature for 5 minutes, and then a mixture of Rho and ATP was added and incubated at 35°C. Mean values of three independent experiments were plotted (error bars = S.D.) (Table S2).

(D) RNA release assay with GreA. The bead-immobilized EC was prepared without GreA. GreA (2 pmol per sample) or buffer was added to the EC and incubated at room temperature for 5 minutes, and then a mixture of Rho and ATP was added and incubated at 35°C. Mean values of three independent experiments were plotted (error bars = S.D.) (Table S2).

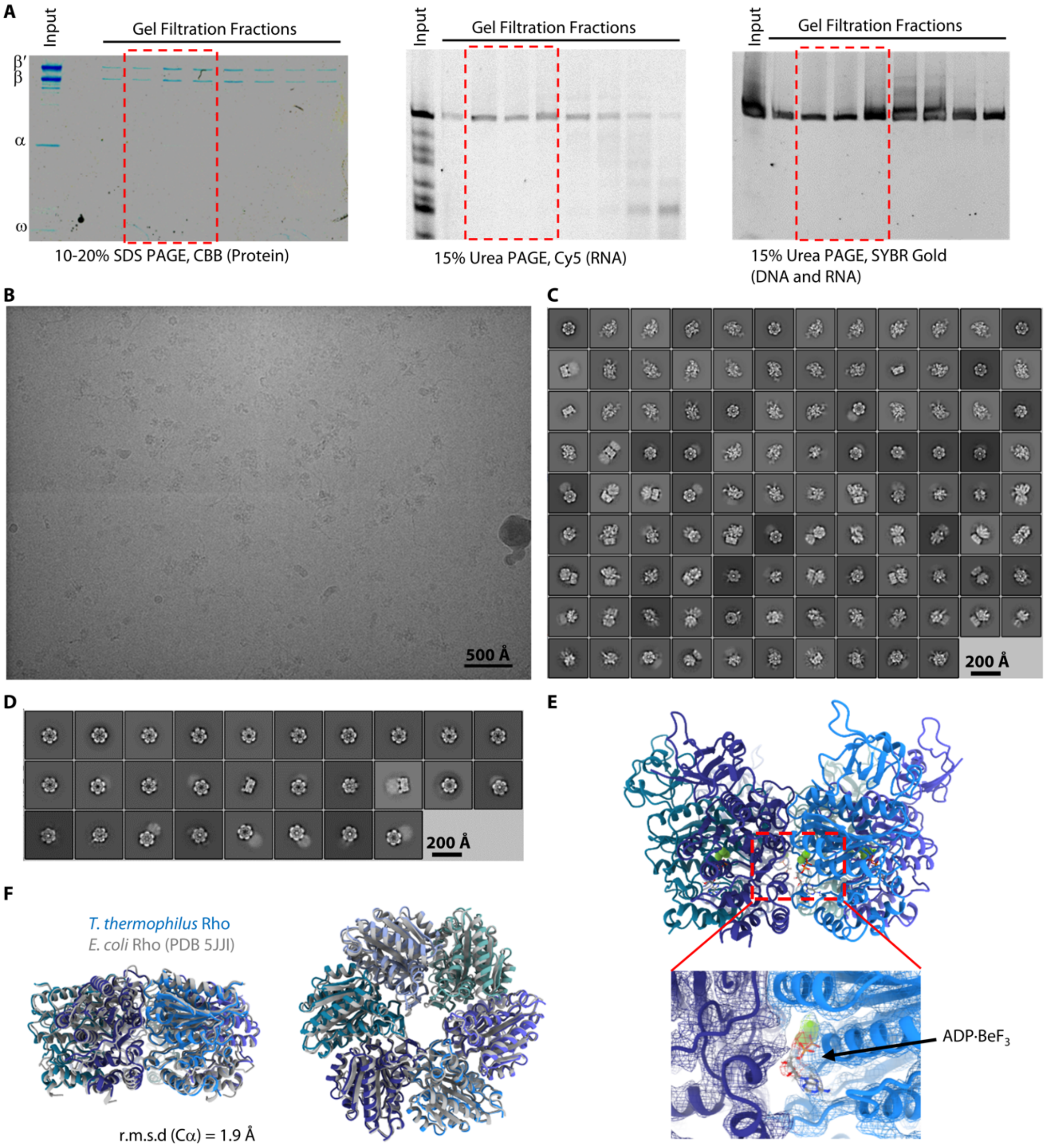

Fig. S2. Sample preparation and image analyses of the RNAP-Rho complex.

(A) Electrophoretic analyses of gel filtration fractions: proteins were detected with SDS-PAGE and DNA/RNA were detected with Urea-PAGE. Center and right are identical Urea-PAGE gels: after detection of fluorescent RNA (center), DNA and RNA were detected by staining gels with SYBR Gold (right). Fractions marked with red boxes were collected for cryo-EM sample preparation.

(B) A raw micrograph of a cryo-EM grid used for this study.

(C) 2D class averages for the RNAP and RNAP-Rho complex particles.

(D) 2D class averages for the Rho-hexamer particles.

(E) A close-up view of ADP·BeF_3 bound between Rho protomers A and B. The ADP is shown in a stick model, and the beryllium and fluoride atoms are shown as spheres. The density map is shown as mesh colored to match the model.

(F) Superimposition of *T. thermophilus* Rho and *E. coli* Rho in the closed-ring form (PDB 5JJI). The models were superimposed by their CTD regions (Tth: residues 140-420, Eco: residues 131-413).

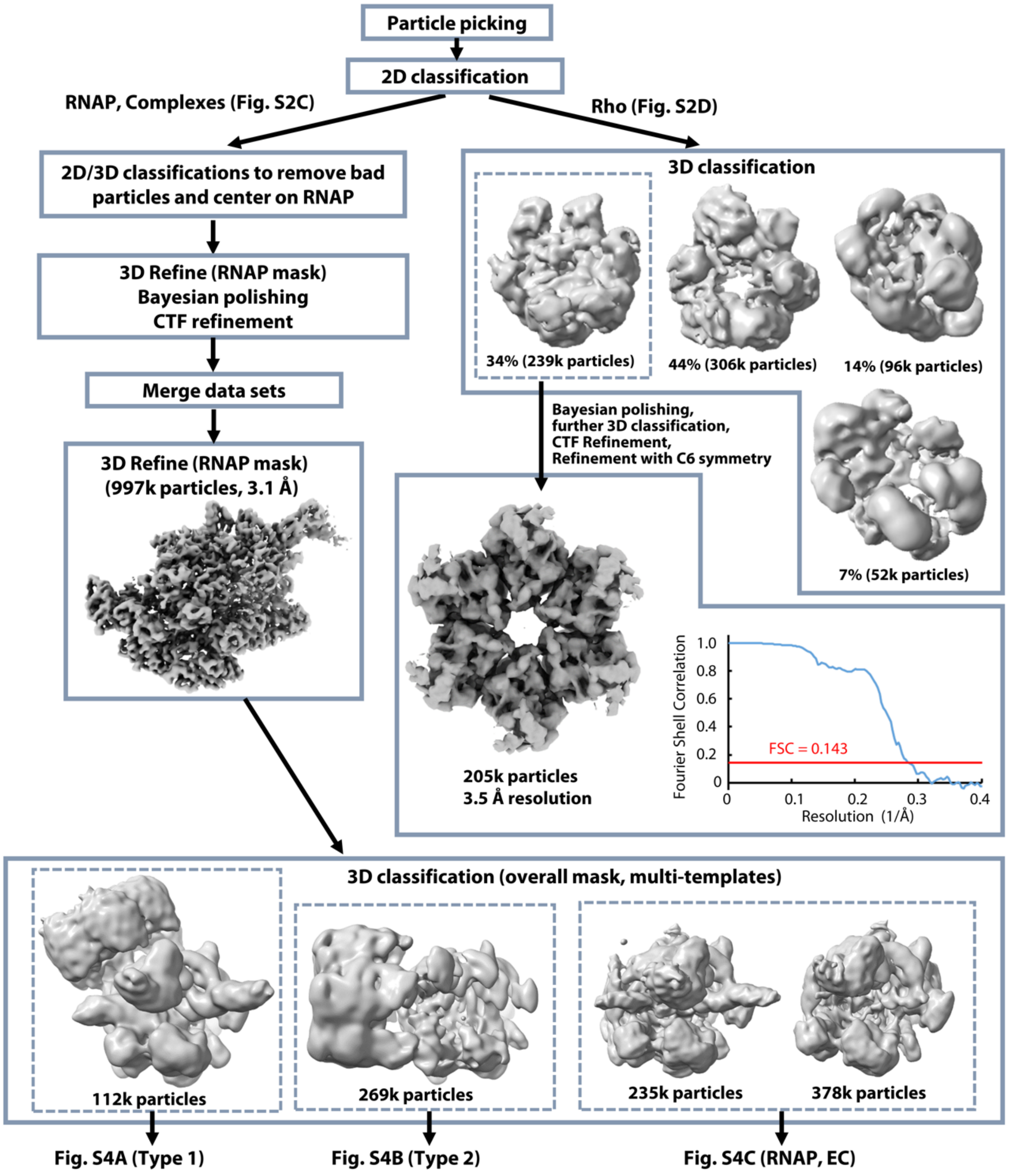

Fig. S3. Workflow of the image analysis.

Workflow of single-particle image analysis. Further analyses of particles containing RNAP are shown in Fig. S4.

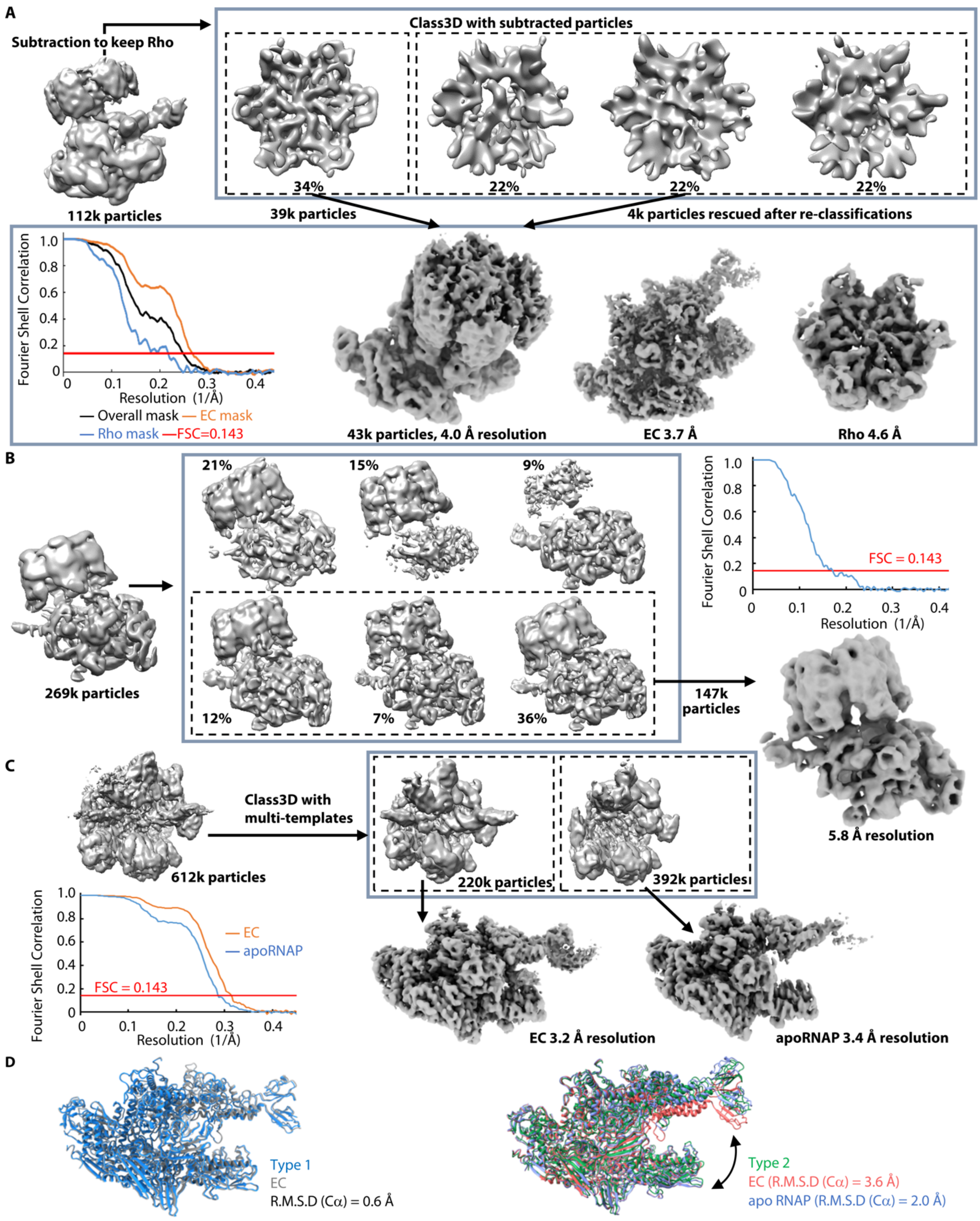

Fig. S4. Workflow of the image analysis and comparison of RNAP conformations in different complexes.

(A) Image analysis of the type-1 complex.

(B) Image analysis of the type-2 complex.
(C) Image analysis of EC and apo RNAP.
(D) Comparison of RNAP conformations. Left: RNAP in the type-1 complex is superimposed with that in the EC by the RNAP core module (the two α subunits, residues 1-17, 394-700, 833-997 of the β subunit, and residues 781-1069 of the β′ subunit). Right: RNAP in the type-2 complex is superimposed with apo RNAP and EC by the RNAP core module.

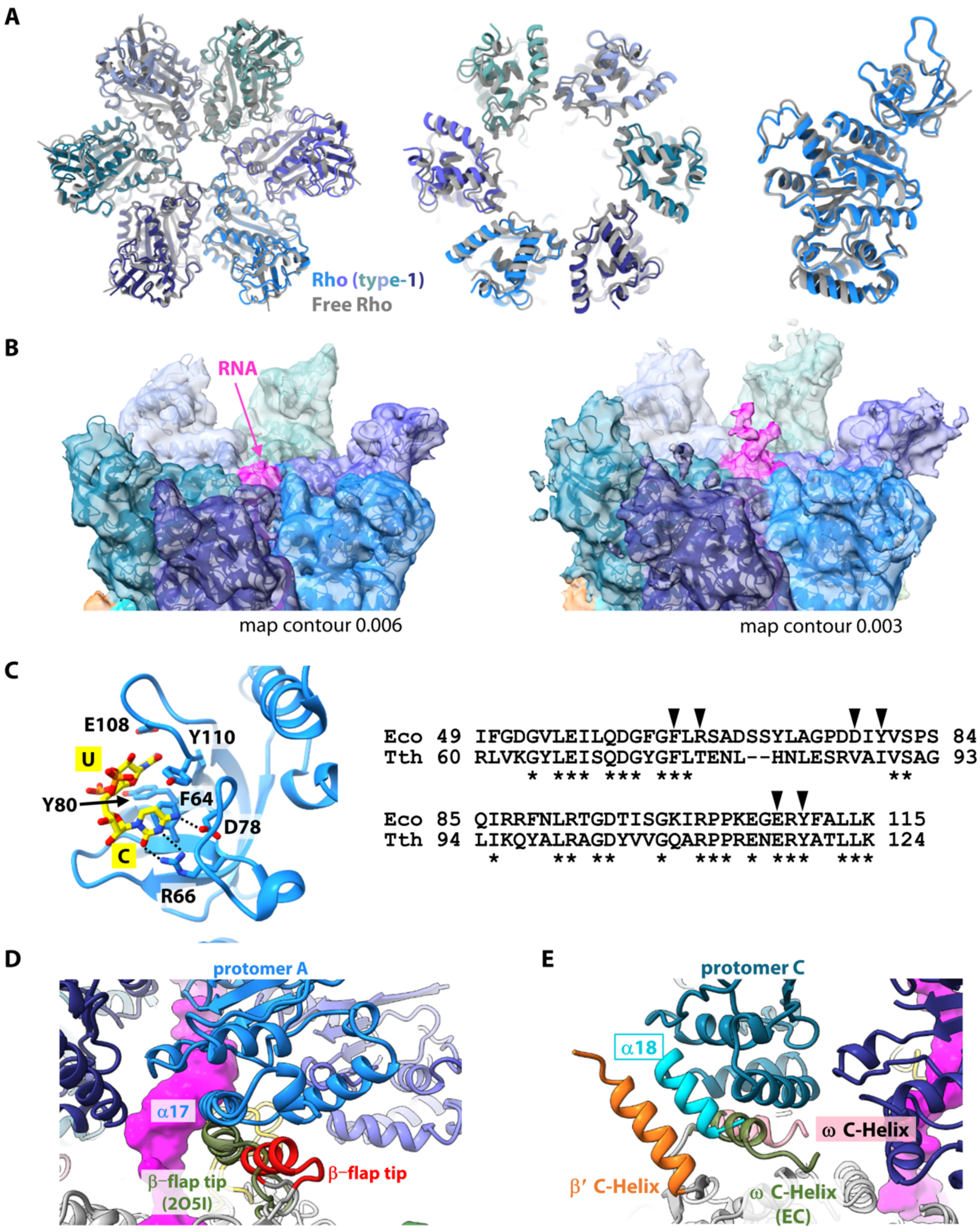

Fig. S5. Structure of the type-1 complex.

(A) Comparison of the Rho conformation in the type-1 complex with the RNAP-free Rho.
(B) Cryo-EM maps of the Rho-NTD in the type-1 complex. Maps with different contours are shown. RNA is colored magenta.
(C) Left: Recognition of pyrimidine nucleotides by the PBS in *E. coli* Rho (PDB 1PVO) *(18)*. Right: Sequence alignment of the PBS regions in *E. coli* and *T. thermophilus* Rho. Residues that directly recognize pyrimidine nucleotides in *E. coli* are indicated by arrows.

(D) Conformational change of the β-flap tip in the type-1 complex. The crystal structure of *T. thermophilus* EC (PDB 2O5I) (*32*) is superimposed on the type-1 complex by the β-flap domain (residues β701-832), and part of the β-flap tip (β762-784) is shown. While the type-1 complex is colored as in Fig. 2C, the β-flap tip of the EC (PDB 2O5I) is colored moss green.
(E) Conformational change in the ω subunit C-terminal helix in the type-1 complex. The structure of the Rho-unbound EC (Figs. 1D, S4C) is superimposed on the type-1 complex, and part of the ω C-helix (residues ω81-96) is shown. While the type-1 complex is colored as in Fig. 2C, the ω C-helix part of the Rho-unbound EC is colored moss green.

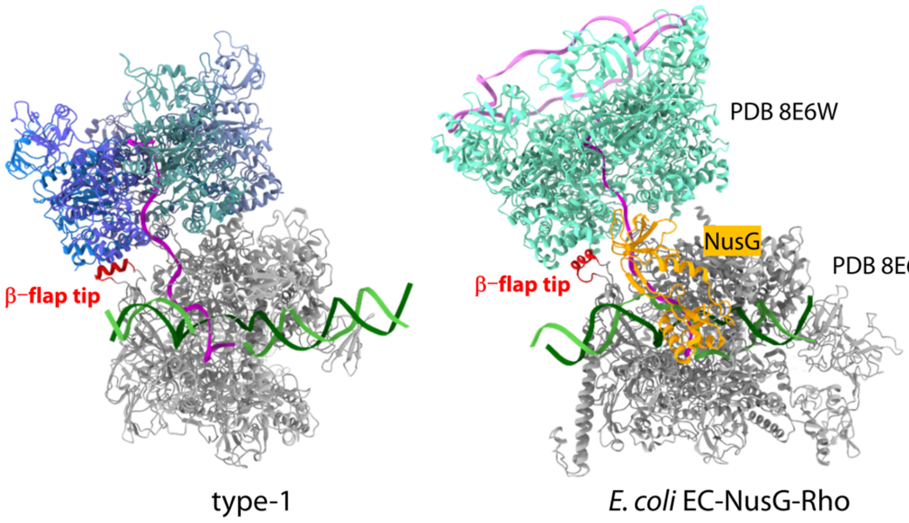

Fig. S6. Comparison with *E. coli* EC-Rho structure.

Structures of the *T. thermophilus* EC-Rho complex (type 1) (left) and the *E. coli* EC-NusG-Rho complex (*33*) (right) are shown for comparison.

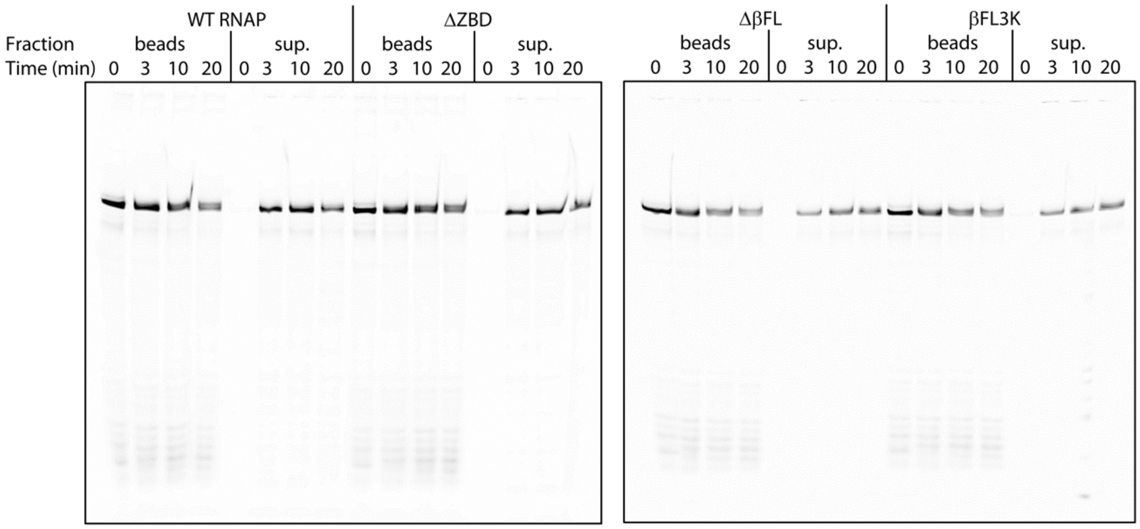

Fig. S7. Gel images for the RNA release assay with RNA variants.

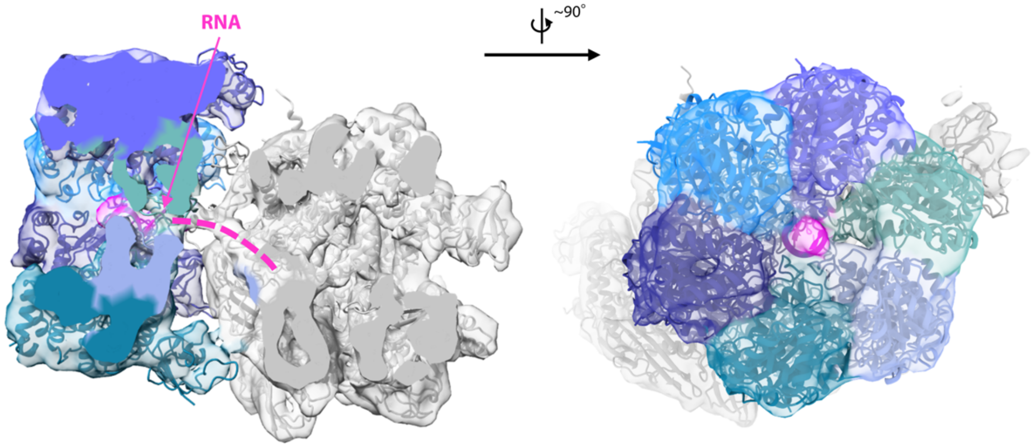

Fig. S8. RNA density in the type-2 complex.

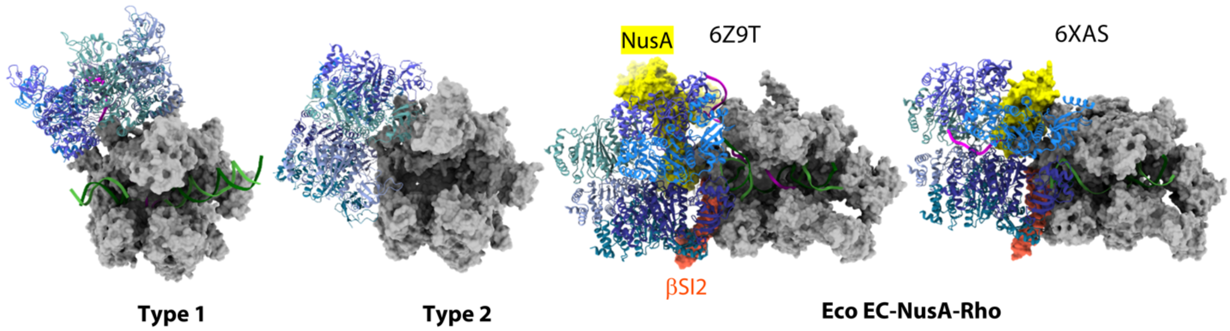

Fig. S9. Comparison with *E. coli* EC-NusA-Rho complex structures.
T. thermophilus EC-Rho complexes (types-1 and -2) and the *E. coli* EC-NusA-Rho complex structures (*25, 26*) are shown for comparison.

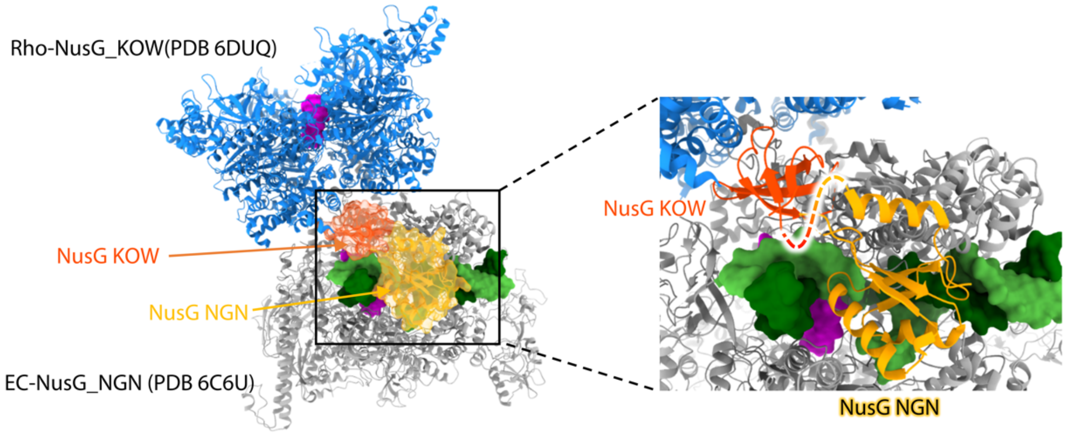

Fig. S10. Model of NusG binding.

The N-terminal (NGN) domain of NusG in the *E. coli* EC-NusG complex (PDB 6C6U) (*36*) was superimposed on the type-1 complex by the β′ coiled-coil (Tth: residues 539-583, Eco: residues 264-308). The C-terminal (KOW) domain of NusG in the *E. coli* Rho-NusG KOW domain complex (PDB 6DUQ) (*35*) was superimposed on the type-1 complex by the CTD of Rho protomer F (Tth: residues 140-420, Eco: residues 131-413). The linker between the N- and C-terminal domains is depicted as a dotted line.

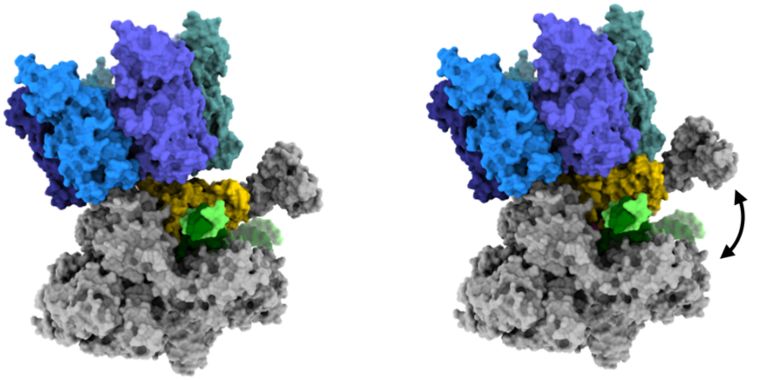

Fig. S11. Modeling of clamp opening in the type-1 complex.

Left: type-1 complex; Right: apo RNAP was superimposed on the type-1 complex by the shelf module of RNAP (residues β 1006-1080, β′ 621-782 and β′ 1103-1430).

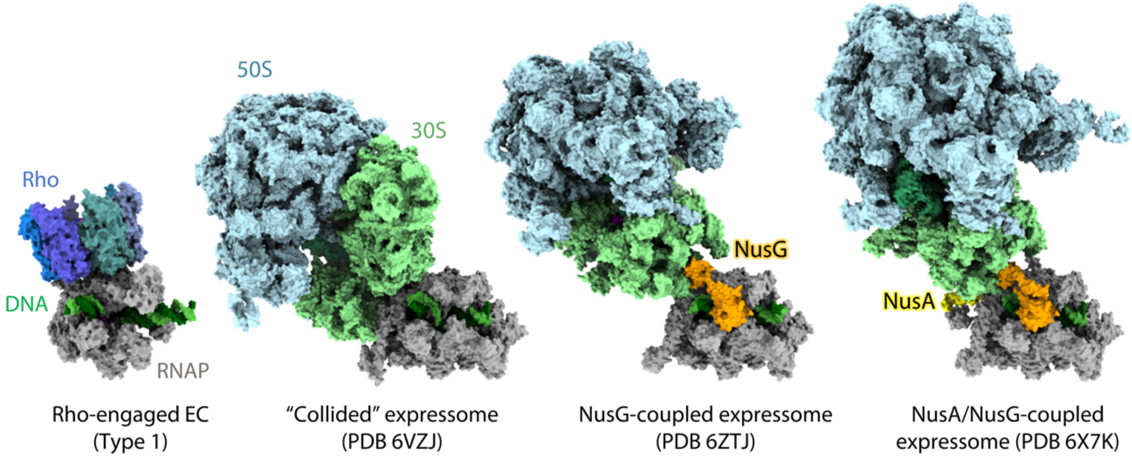

Fig. S12. Overlapped binding sites of Rho and the ribosome on the EC.

Table S1. Data Collection and image processing.

Data collection and processing

	Dataset 1	Dataset 2
Number of micrographs	13,764	19,776
Magnification	81,000	
Voltage (kV)	300	
Pixel size (Å)	1.06	
Electron exposure (e–/Å^2)	50	
Defocus range (μm)	-0.9 ~ -2.7	

Refinement

	Rho	EC	apo RNAP	Type 1			Type 2
No. particle images	205,226	219,822	392,560	43,245			147,189
				composite	EC-mask	Rho-mask	
Map resolution* (Å)	3.5	3.2	3.4	4.0	3.7	4.6	5.8
CC mask	0.65	0.78	0.79	0.60	0.82	0.62	0.58
CC volume	0.64	0.75	0.79	0.68	0.81	0.66	0.61
Model composition							
Non-hydrogen atoms	17,244	25248	23072	42819			40492
Protein residues	2,166	2971	2923	5143			5093
DNA/RNA residues	0	87	0	101			7
Ligands	18	3	3	21			21
R.m.s. deviations							
Bond lengths (Å)	0.004	0.003	0.004	0.005			0.007
Bond angles (°)	0.749	0.658	0.699	0.782			0.977
Validation							
MolProbity score	2.52	2.01	2.1	2.29			4.08
Clashscore	16.35	12.45	15.21	22.81			132.26
Poor rotamers (%)	4.93	0.04	0.08	0.02			17.18
Ramachandran plot							
Favored (%)	95.96	94.01	93.87	93.1			89.69
Allowed (%)	3.71	5.95	6.13	6.74			9.99
Disallowed (%)	0.32	0.03	0	0.16			0.32
PDB ID	8HSJ	8HSG	8HSH	8HSR	-	-	8HSL
EMDB ID	34999	34996	34997	35004	-	-	35000

* FSC threshold = 0.143

Table S2. Data for RNA release assays

RNAP variant	Rho variant	Additional factor	% RNA released				related to
			0 min.	3 min.	10 min.	20 min.	
WT	WT	-	1.35	43.60	47.68	47.96	Fig. 3B
WT	WT	-	0.44	48.06	58.95	52.24	Fig. 3B
WT	WT	-	0.17	38.87	51.78	49.94	Fig. 3B
ΔZBD	WT	-	0.33	41.61	50.68	43.90	Fig. 3B
ΔZBD	WT	-	0.22	47.99	61.64	55.16	Fig. 3B
ΔZBD	WT	-	1.79	50.21	60.57	45.53	Fig. 3B
ΔβFL	WT	-	0.24	30.47	46.27	54.48	Fig. 3B
ΔβFL	WT	-	0.14	29.25	41.20	51.33	Fig. 3B
ΔβFL	WT	-	0.48	26.23	45.32	55.28	Fig. 3B
βFL3K	WT	-	0.69	29.02	38.61	53.70	Fig. 3B
βFL3K	WT	-	0.17	28.82	42.22	51.13	Fig. 3B
βFL3K	WT	-	0.40	27.72	38.59	48.63	Fig. 3B
WT	WT	-			32.05		Fig. 4C
WT	WT	-			43.40		Fig. 4C
WT	WT	-			42.64		Fig. 4C
WT	T4L70	-			41.16		Fig. 4C
WT	T4L70	-			45.30		Fig. 4C
WT	T4L70	-			43.35		Fig. 4C
WT	T4L115	-			42.84		Fig. 4C
WT	T4L115	-			48.40		Fig. 4C
WT	T4L115	-			46.28		Fig. 4C
WT	T4L426	-			20.14		Fig. 4C
WT	T4L426	-			22.90		Fig. 4C
WT	T4L426	-			19.84		Fig. 4C
WT	WT	-	1.18	37.22	51.15	58.84	Fig. S1C
WT	WT	-	0.64	41.31	54.76	57.09	Fig. S1C
WT	WT	-	1.09	40.96	50.33	58.68	Fig. S1C
WT	WT	NusG	1.54	39.07	55.67	58.80	Fig. S1C
WT	WT	NusG	0.84	46.28	54.00	59.13	Fig. S1C
WT	WT	NusG	0.45	45.53	57.56	64.34	Fig. S1C
WT	WT	-	0.34	43.14	50.65	43.07	Fig. S1D
WT	WT	-	0.28	37.52	49.85	42.43	Fig. S1D
WT	WT	-	0.36	42.41	54.06	39.26	Fig. S1D
WT	WT	GreA	0.37	44.75	54.85	44.08	Fig. S1D
WT	WT	GreA	0.61	44.59	58.25	40.01	Fig. S1D
WT	WT	GreA	0.96	42.07	58.69	47.17	Fig. S1D

Movie S1. Structure of the Rho-engaged RNAP EC.
